# Timescales of Multineuronal Activity Patterns Reflect Temporal Structure of Visual Stimuli

**DOI:** 10.1371/journal.pone.0016758

**Published:** 2011-02-08

**Authors:** Ovidiu F. Jurjuţ, Danko Nikolić, Wolf Singer, Shan Yu, Martha N. Havenith, Raul C. Mureşan

**Affiliations:** 1 Department of Neurophysiology, Max Planck Institute for Brain Research, Frankfurt, Germany; 2 Department of Experimental and Theoretical Neuroscience, Center for Cognitive and Neural Studies (Coneural), Romanian Institute of Science and Technology, Cluj-Napoca, Romania; 3 Department of Neuroscience, Frankfurt Institute for Advanced Studies (FIAS), Frankfurt, Germany; Indiana University, United States of America

## Abstract

The investigation of distributed coding across multiple neurons in the cortex remains to this date a challenge. Our current understanding of collective encoding of information and the relevant timescales is still limited. Most results are restricted to disparate timescales, focused on either very fast, e.g., spike-synchrony, or slow timescales, e.g., firing rate. Here, we investigated systematically multineuronal activity patterns evolving on different timescales, spanning the whole range from spike-synchrony to mean firing rate. Using multi-electrode recordings from cat visual cortex, we show that cortical responses can be described as trajectories in a high-dimensional pattern space. Patterns evolve on a continuum of coexisting timescales that strongly relate to the temporal properties of stimuli. Timescales consistent with the time constants of neuronal membranes and fast synaptic transmission (5–20 ms) play a particularly salient role in encoding a large amount of stimulus-related information. Thus, to faithfully encode the properties of visual stimuli the brain engages multiple neurons into activity patterns evolving on multiple timescales.

## Introduction

Neuronal coding is a central issue in the investigation of brain function [1], [2] and has both a spatial and a temporal aspect. The spatial aspect refers to distributed coding across multiple neurons in the cortex, while the temporal aspect pertains to the timescale of this process. Information coding in the brain has been mostly investigated such that either one or the other of these two aspects has been neglected.

Due to inherent technical difficulties, the spatial aspect of coding was mostly ignored in early studies. These focused on single-electrode recordings [3], neurons being probed independently, i.e., one-by-one. Information about distributed coding was not accessible to these techniques, such that the most salient property of single-electrode signals was considered to be the firing rate [3], [4]. With the advent of multi-electrode recordings, distributed coding started to be more thoroughly investigated [5] but issues related to timescale were not systematically explored. Most reports have focused either on a very fast timescale (<20 ms), i.e. spike-synchrony [5]–[9] or have investigated only a limited range of timescales. For example, in their multivariate analysis, Friedrich and Laurent [10] used sliding windows of 400 ms studying ensemble coding in the zebrafish olfactory system. A similar strategy was employed by Brown and Stopfer in the locust olfactory system [11], on timescales of 50 ms and 100 ms. Bathellier et al. [12] used windows on the order of 40 ms and >100 ms and studied coding in the mouse olfactory bulb.

A more systematic study of timescales relevant for coding was undertaken by Butts et al. [13], but only for the case of single cells in the LGN. For populations of neurons, a recent study reported spike-timing precision in the LGN on the order of ∼10 ms in responses to natural scenes [14]. This study however did not explicitly focus on how the encoding of stimulus features comes about, and did not investigate how the temporal properties of different stimuli may be reflected in the timescale of neuronal responses.

For the case of multiple neurons we do not yet have clear and complete characterizations of how multineuronal activity patterns contribute to coding. Recently, the ability of computer-simulated readout neurons to extract stimulus-related information from distributed neuronal activity was systematically investigated for multiple timescales [15]. Results revealed a surprising degree of relevance of short timescales (< = 20 ms) for sequences of briefly flashed high-contrast images. This study used only one type of stimulus and left it open whether a similar result would be obtained with stimuli having different temporal properties.

An exploration of multineuronal activity, systematically covering a broad range of timescales and different types of stimuli is still missing. Especially relevant are aspects that refer to the timescale of patterns evoked by stimuli with various temporal properties and aspects that refer to the stimulus time-locking of these patterns. One major limitation impairing such investigations is the scarcity of analysis methods able to cope with the simultaneous behavior of multiple neurons [16], [17] over multiple timescales. Furthermore, conceptually different analysis techniques are usually employed to investigate the presence of slow and fast codes. We argue that it is necessary to investigate coding across multiple neurons and multiple timescales in a unified manner, by employing always a conceptually identical method. In addition, because natural visual stimuli have a broad range of temporal properties [13], the neuronal code may need to be explored with a set of stimuli that exhibit a similar variety of temporal dynamics.

Here, we matched these important requirements by recording responses in cat primary visual areas to stimuli that changed either with a slow rhythm (drifting sinusoidal gratings), fast rhythm (high-contrast stimuli flashed in fast sequences; 100 ms duration and 100 ms inter-stimulus-interval), or had a mixture of fast and slow epochs (movies of natural scenes). We then applied a recently introduced analysis method that is able to detect and visualize evolution of multineuronal cortical firing patterns on an arbitrary timescale [18]. In particular, we investigated how multineuronal activity patterns emerged in the visual cortex with respect to timescale and stimulus-locking, and the degree to which the contributions of fast, intermediate, and slow coding mechanisms changed as a function of temporal properties of stimuli.

## Results

### From Neurons to Patterns

In a previous report [18] we have described a method to detect stereotypically appearing activity patterns in a set of multineuronal spike-trains. The method first transforms multiple spike-trains by convolution with exponentially decaying kernels [19], [20] (low-pass filtering). Convolution enables the manipulation of the timescale of interest through the decay (integration) time constant (*τ*). Small time constants (*τ* = 1–5 ms) correspond to constellations of coincident spikes (synchronous spikes/joint-spike events) [21], while large time constants (*τ*>100 ms) extract collective firing-rate modulations.

**Figure 1:**
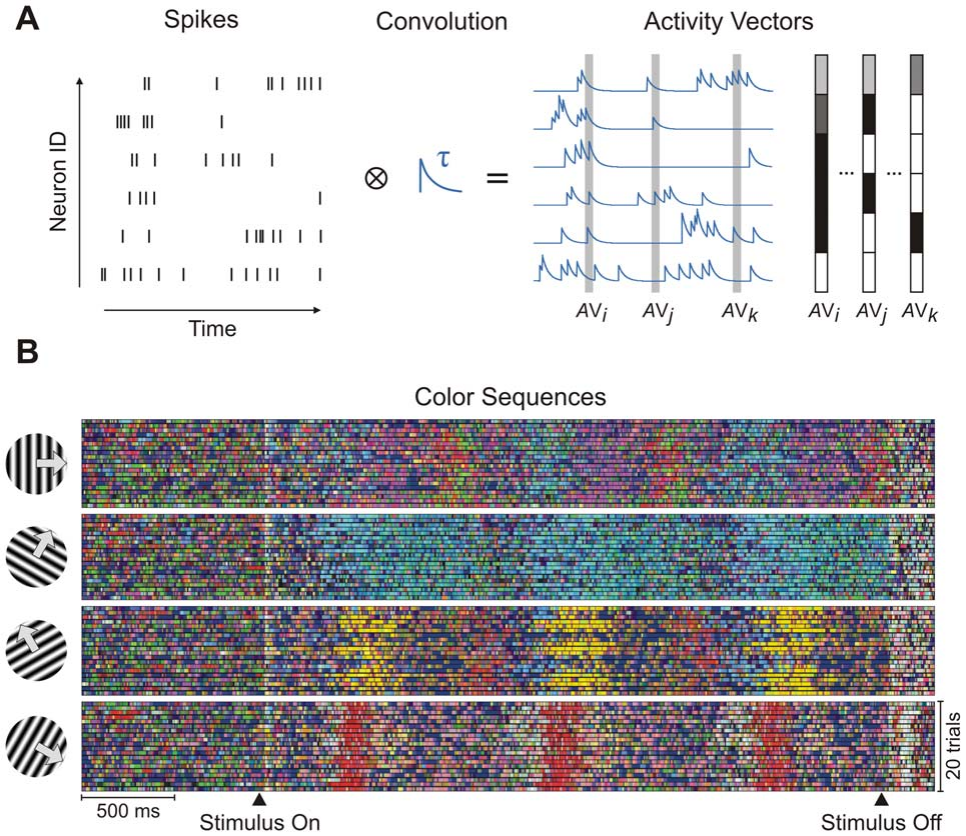
From spikes to multineuronal patterns. (A) Low-pass filtering of simultaneously recorded spike trains by convolution with a decaying exponential function. Activity vectors are obtained by sampling the resulting continuous traces at each time step. (B) Color representation of stereotypical patterns corresponding to activity vectors from a dataset recorded with drifting sinusoidal gratings. Color sequences corresponding to trials evoked by 4 grating stimuli are shown, grouped by the stimulus (*τ* = 20 ms).

After low-pass filtering by convolution (Figure 1A) the multiple continuous traces were sampled [22] with a frequency of 1 kHz, defining *activity vectors* that were clustered using a three-dimensional (3D) Kohonen map [23]. The resulting clusters approximate classes of stereotypically appearing activity vectors, which are called *model vectors* [23], [24] and are representative for a given dataset. For simplicity and readability we will subsequently refer to model vectors as *patterns*. We constructed *model trials* by replacing the activity vectors with their corresponding model vectors (patterns). The subsequent analyses were then based on model trials and therefore all results on patterns refer to the properties of model vectors (obtained after clustering). The use of an ordered clustering algorithm, such as a 3D Kohonen map, enables also the visualization of multineuronal activity patterns through colors, whereby each appearing pattern is represented by a single line of a corresponding color. The succession of patterns along a trial can then be represented by a horizontal sequence of colored lines (color sequence) [17]. Most importantly, color sequences from multiple trials can be shown adjacent vertically to provide an overall picture about the repeatability of patterns. Typically, each stimulus produces its own set of patterns (Figure 1B) [17]. If visualization is not required (ordered mapping is not needed), any clustering algorithm can in principle replace the 3D Kohonen map. For the data presented here we found that results with Kohonen clustering were very similar to those obtained with K-Means or LBG (Linde-Buzo-Gray) [25] clustering (see Considerations Regarding Clustering in Text S1, Figures S1 and S2, and section A Collective Code on Multiple Timescales below). Next we investigated how information was encoded by neuronal patterns on different timescales.

### Integration Constant and Time-Locking to Stimulus

**Figure 2:**
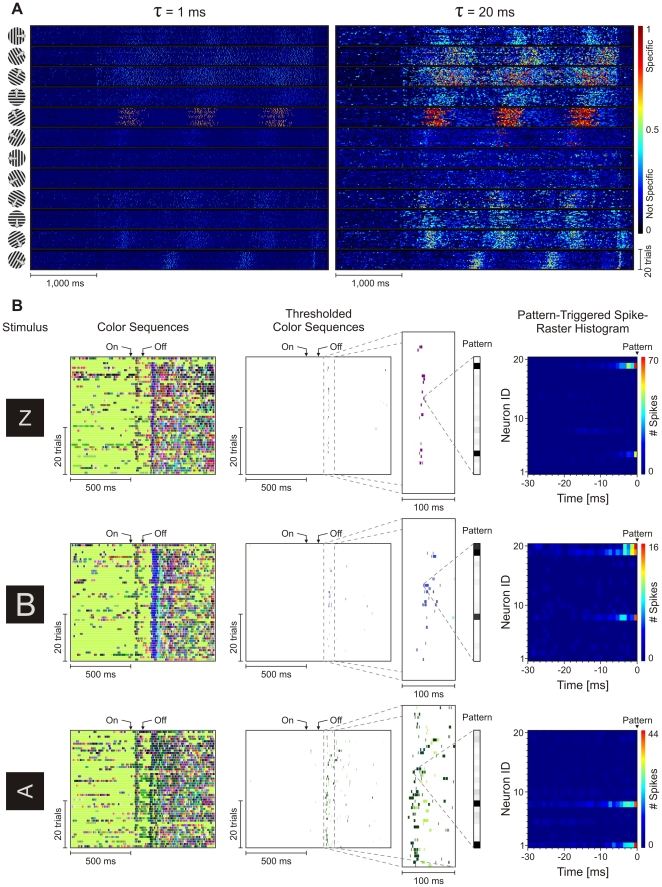
Pattern specificity. (A) Specificity plots at two integration time constants for responses to drifting sinusoidal gratings. Far right: color intensity code for specificity. (B) Appearance of specific and stimulus-locked patterns with a time constant of 5 ms. Three examples (rows) are shown from the dataset with 49 flashed graphemes. From left to right: stimulus, color sequences on 50 trials, specificity thresholded color sequences (see text) with inset showing patterns precisely stimulus-locked across trials, pattern-triggered spike raster histograms (see text). Activation of component neurons in the patterns is shown in the second level inset (“Pattern”) with grayscale coding (white, activation  = 0; black, activation ≥1).

We first tested whether stimulus related information carried by patterns (i.e., stimulus specificity of patterns) depends on the choice of the timescale at which patterns are investigated. In addition, we tested whether the timing of patterns was locked to the timing of the stimulus and the degree to which this locking depended on the type of stimulus. A measure of stimulus-specificity for a pattern, called *pattern specificity*, was defined as a quantity representing the estimated probability that a pattern appears for a particular stimulus from a set of stimuli [18]. A pattern with high specificity for a stimulus allows one to discriminate that stimulus from other stimuli of the set. By manipulating the integration constant and computing the specificity of patterns, one can identify the optimal timescale on which information is best encoded, i.e., the optimal timescale for a given set of stimuli. Figure 2A depicts specificity of patterns computed with two integration time constants (1 ms and 20 ms) for responses evoked with drifting sinusoidal gratings. Patterns evolving on a timescale of 20 ms had higher stimulus specificity than those on 1 ms, indicating that stimulus-related information was encoded more accurately on the former than on the latter timescale. Also, patterns with high specificity were not precisely stimulus-locked across trials (in the millisecond range), but stimulus locking was broad, comparable to the slow modulation induced by the grating.

Very different results were obtained when stimuli were briefly flashed on the screen. Our second set of responses was obtained by flashing 49 individual graphemes for 100 ms [26], [15] and recording the activity of 20 neurons simultaneously. In the example color sequences [18] in Figure 2B a small integration time constant of 5 ms was sufficient for specific patterns to carry stimulus-related information on this fast timescale. The results are shown for three graphemes (a “Z”, an “enlarged B” and a “rotated A”). The onset-responses had a short latency (30–40 ms) and were transient, while the offset-responses had a latency of 70 ms and were much more sustained [26], [15] (Figure 2B, color sequences). Pattern specificity was assessed by comparing responses over the entire set of 49 graphemes and only the patterns with a specificity >0.25 are shown in the middle column of Figure 2B (Thresholded Color Sequences). [18]. This high threshold (chance level: 1/49 = 0.02) isolates well the most specific pattern for stimulus “Z”. With lower specificity thresholds, gradually more patterns can be revealed, which carry progressively less information about stimuli. The most specific pattern for stimulus “Z” occurred only within a narrow temporal window of <17 ms (Figure 2B, Thresholded Color Sequences, top, inset) in 16 out of 50 trials. For “enlarged B”, a specific pattern occurred in a window <40 ms, in 15 out of 50 trials (Figure 2B, Thresholded Color Sequences, middle, inset). The “rotated A” stimulus elicited a specific pattern in 38 out of 50 trials but in a broader temporal window and in association with a few other patterns (Figure 2B, Thresholded Color Sequences, bottom, inset). Relative to the variability of cortical responses [27], the temporal precision of the first occurrence of each pattern was rather high: The range of jitter (min-max) across trials was ∼15 ms for “Z”, ∼23 ms for “enlarged B”, and ∼21 ms for “rotated A”. We also computed pattern-triggered spike-raster histograms (PTSRH) [18], i.e. for occurrences of the pattern at times *t_i_*, we summed the spike rasters (1 =  spike; 0 = no spike) corresponding to 30 ms windows before each *t_i_* (Figure 2B, Pattern-Triggered Spike-Raster Histogram). PTSRHs revealed that different neurons were active corresponding to different patterns and that spikes contributing to patterns frequently participated in bursts: Spikes were often preceded by other spikes of the same neurons, at inter-spike intervals <8 ms [28]. PTSRHs were also consistent with the activations in the patterns (Figure 2B, “Pattern” inset), showing that patterns (model vectors) computed by the clustering algorithm reflected very closely the real spiking constellation.

These examples in Figures 2A and 2B suggest that stimulus specific patterns may be expressed on different timescales, may occur at various moments in time, and the precision with which they are locked to the stimulus may vary.

### A Collective Code on Multiple Timescales

To investigate systematically the importance of timescales for stimulus coding by patterns we applied three classification strategies (i.e. types of classifiers), each relying on different features of neuronal activity. All of them integrated the information available over the entire duration of a trial and were always trained on one half of the trials (training set; randomly chosen), while the classification performance was tested on the other half (testing set). The three classifiers relied on the following features, respectively: combinations of mean firing rate (*mean rate classifier*), the specificity of patterns irrespectively of where in the trial they occur (*specificity classifier*), and the time-specific position of the patterns within the trial (*trajectory classifier*). The specificity classifier ignored the stimulus-locking of patterns, while the trajectory classifier was strongly dependent on stimulus-locking (see Materials and Methods and Classification in Text S1). The performance of these classifiers was tested on responses to three types of stimuli: slow sinusoidal gratings, natural movies with mixture of speeds, and briefly flashed letter sequences (sequences of three letters, each similar to those in Figure 2B – flashed for 100 ms with a 100 ms inter-letter-interval; see Materials and Methods). In a first analysis, patterns were computed only with *τ* = 20 ms and trials were randomly assigned 1,000 times into training and testing sets.

**Figure 3:**
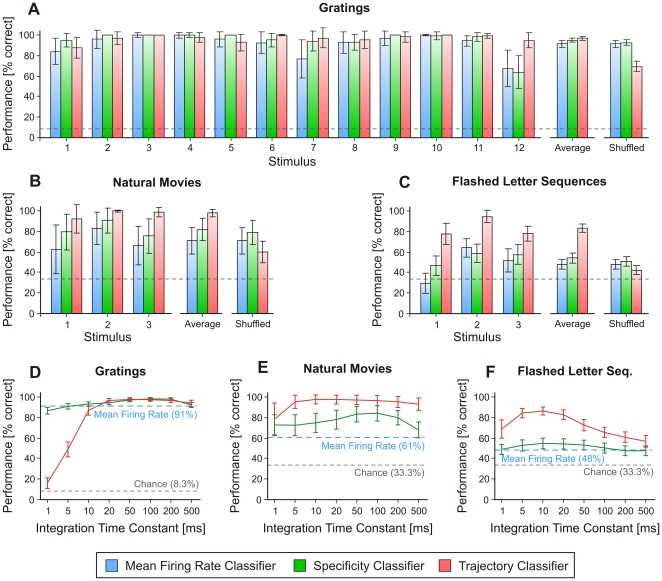
Information content of patterns and dependence of classification on the temporal scale. (A)–(C) Classification performance of three classifiers with τ = 20 ms (blue: mean rate; green: pattern specificity; red: pattern trajectory) for datasets evoked with drifting sinusoidal gratings (A), natural movies (B), and flashed letter sequences (C). Shown are: performance for each stimulus condition, average performance, and average performance after shuffling the spike-trains (see text). (D)–(F) Performance is shown as a function of the integration time constant (*τ*) for datasets with drifting sinusoidal gratings (D), natural movies (E), and flashed letter sequences (F). Error bars represent s.d. Dashed lines mark chance levels.

For drifting sinusoidal gratings, all three classifiers performed with high accuracy (on average, >90% correct classifications), with a slightly higher average performance of the trajectory classifier (97% correct vs. 91% and 95% for mean rate and specificity classifiers, respectively; Figure 3A). For stimuli with natural scenes the trajectory classifier yielded almost perfect discrimination between stimuli (98% correct), outperforming considerably both the mean rate (61%) and the specificity (78%) classifiers (Figure 3B). For flashed letter sequences (Figure 3C), the trajectory classifier had also highest accuracy (83% correct), which was well above that of the mean rate and specificity classifiers (48% and 53% correct, respectively). Thus, overall, the classifier relying on mean firing rate was the least accurate and the one relying on trajectories the most effective for distinction between the stimuli.

To investigate the importance of the location of patterns along the trial, we segmented trials in 20 ms windows that were then randomly permuted (shuffled) for each trial independently. Kohonen maps were reconstructed and classifiers were re-applied. As expected, classification performance was reduced only for the trajectory classifier (Figures 3A–C, shuffled, red), in agreement with this classifier's sensitivity for stimulus-locked sequences of patterns. Nevertheless, the performance stayed above chance even for the shuffled data, and was especially high for gratings and natural movies (Figures 3A and 3B). This result can be explained by redundant expression of some patterns along the trials, which are especially likely to occur during, e.g., the repeated passes of the bars of gratings. In such cases, a permutation of two similar spiking windows leads to little reduction in the final classification of the permuted data.

The dependence of classification performance on the timescale of patterns was then explored by manipulating the integration time constant, re-computing Kohonen maps for each time constant, and reclassifying all the datasets. For drifting sinusoidal gratings, the performance of the specificity classifier was high across the whole range of explored time constants (Figure 3D, green line). The trajectory classifier also exhibited high performance for most values of *τ* with the exception of those <10 ms (Figure 3D, red line). The difference between the two pattern classifiers on fast timescales (1–5 ms) suggests that specific patterns evolving on these timescales are not precisely time-locked to stimulus (see Classification in Text S1). This result is consistent with results reported in Figure 2A for the same stimuli.

For natural movies (Figure 3E), the trajectory classifier outperformed the mean rate and the specificity classifier for all values of *τ*, the difference being largest for time constants of 5–20 ms (Figure 3E). These findings indicate the locking of brain dynamics to both fast and slow events in the stimuli, consistent with the rich temporal structure of natural movies.

Finally, flashed letter sequences entrained stimulus-specific responses almost exclusively on fast timescales. Both the specificity and the mean rate classifiers performed poorly (Figure 3F) in comparison to the trajectory classifier. The performance of the latter peaked at time constants of 10 ms, replicating the results obtained by applying a different analysis method to the same data [15], [26]. Hence, for flashed letter sequences, information about stimulus identity was best encoded in the temporal sequence of stimulus-locked, fast patterns (evolving on timescales of ∼10 ms). We reproduced the reported classification results also on additional datasets and animals (see Figures S3 and S4 and Consistency of Classification Results in Text S1).

### Jittering tests

**Figure 4:**
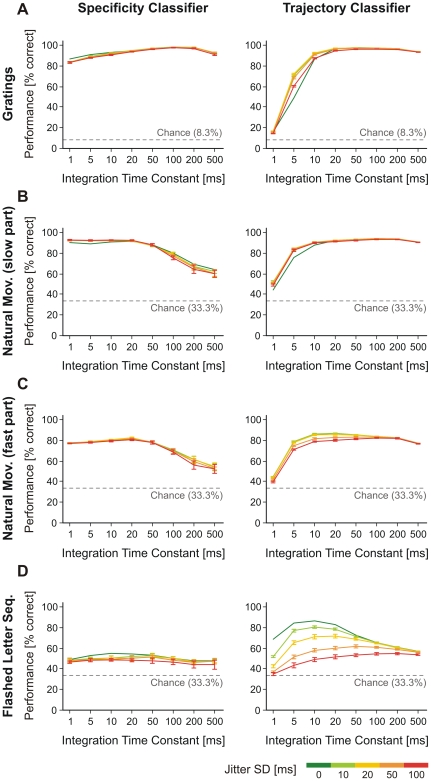
Effect of spike jitter on classification performance. Specificity classifiers (left) and trajectory classifiers (right) for: grating stimuli (A), slow (B) and fast (C) segments of natural movies, and flashed letter sequences (D). The applied jitters are 10 ms (light green), 20 ms (yellow), 50 ms (orange), and 100 ms (red). Original classification performance, without jittering, is shown in dark green curves. Error bars represent s.d. over independent jitters.

To test whether the timing of spikes was important on a given timescale for patterns, we applied jittering to the original spike trains. Each spike was jittered independently with a given amount of noise (SD between 0–100 ms) as this is expected to interfere with patterns evolving on timescales smaller than the jitter. Classifications in Figure 3D–F were then recomputed for each magnitude of jitter (Figure 4). To harvest variability due to the jittering procedure only, for each independent jitter we computed the average performance over multiple train/test half-splits of trials and multiple Kohonen maps. For each jitter amplitude variability was then estimated across average performances yielded by different independent jitters. Because movies with natural scenes contained both fast and slow parts, we analyzed separately the movie parts with slow and fast dynamics (i.e., slow and fast camera movement, respectively).

Overall, jitter affected only slightly the performance of specificity classifiers irrespectively of the type of the stimulus (Figure 4A–D, left). Performance decreased by a small amount with jitter, with the exception of segments of natural movies with slow dynamics, where a small increase in performance was observed, for *τ* between 1–10 ms (Figure 4B, left).

Jitter had more extensive effects on the trajectory classifiers, especially at small timescales. Jitter increased classification performance for slow stimuli (gratings and slow parts of natural movies; Figure 4A,B, right) and decreased performance for fast stimuli (fast parts of natural movies and flashed letter sequences; Figure 4C,D, right). The results in Figure 4D are consistent with those obtained on the same data by a different analysis technique [15], [26]. In all reported cases, effects of jitter were significant (*p*<0.001, one-sample location Z-tests). The increase in performance for slow stimuli on fast timescales can be explained by a more uniform spread of spikes that initially formed bursts (when bursts were artificially removed from the data the effect was no longer present – see Figure S5). This uniform spread ensured that information encoded on slow timescales became available also on fast timescales (e.g., by enabling a more reliable estimate of a slow firing rate vector in a narrow time window and thus increasing the signal-to-noise ratio on fast timescales). In addition, the trajectory classifier is also sensitive to stimulus time-locking of patterns because it averages patterns at corresponding locations across multiple train trials to obtain a model trajectory (see Eq. 13 in Materials and Methods). Thus, at small integration time constants (where the window used to average patterns is also small) the lack of stimulus-locking of bursts creates more variable patterns across the train trials at a given trial location. When spikes in bursts are spread locally by jittering, the patterns at a given location will be more similar across trials and this will increase signal-to-noise ratio of the trajectory classifier at small timescales. The drop in classification performance for fast stimuli indicated that information was encoded largely by fast patterns, precisely locked to stimulus (compare Figures 4D left and 4D right; see also Classifiers Explained Intuitively in Text S1).

### Effect sizes

Classification performance is a non-linear procedure that gives a measurement of the ability of a classifier to separate samples from a given set into their true classes, based on a particular feature (e.g., mean rate, specificity of patterns, trajectory in pattern space). The performance of a classifier cannot fully quantify the structure of the feature space (e.g., distance between samples belonging to different classes) and therefore it does not fully reflect the robustness of a given feature in separating samples into classes. When more samples are added to a dataset, classification performance may degrade. One needs to therefore complement classification by a measurement of robustness/discriminability, i.e., a measure that reflects the structure of the feature space. For example, if points in the feature space can be clearly separated, a performance of 100% is obtained, but this happens both when the distance between classes is small and when it is large. A robustness or discriminability measure should quantify how close in the feature space are the points belonging to different classes.

**Figure 5:**
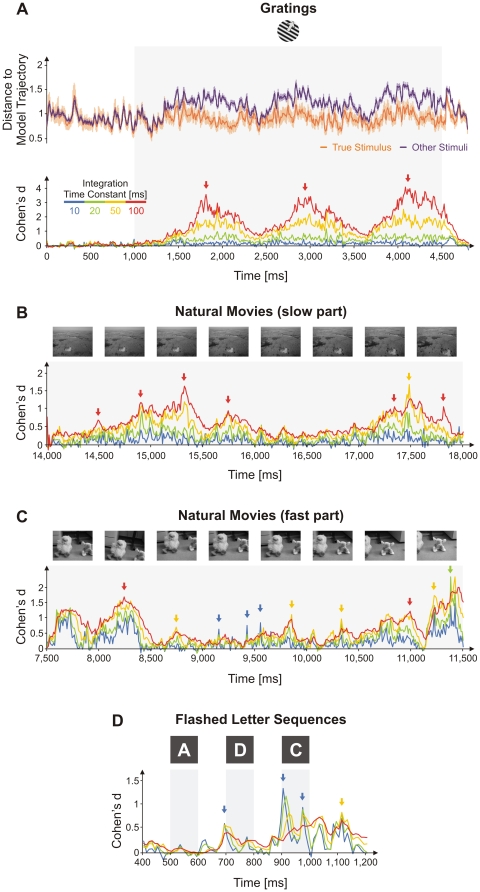
Time of occurrence and timescale of informative patterns. (A) Trajectory analysis on drifting grating stimuli. Top inset: Average distance from trajectories on test trials of a given stimulus to the model trajectory of the true stimulus (orange) and the model trajectories of other stimuli (magenta). *τ* = 20 ms. Cohen's d between the two distance traces for different integration time constants, *τ*, for grating stimuli (bottom inset in (A)), slow (B) and fast (C) segments of natural movies, and a flashed letter sequence (D). Light gray bands indicate stimulus presentation periods. Error bars on distance traces in (A) are s.e.m.

We used a measure of effect size (see Materials and Methods) to quantify, in a time-resolved fashion, the robustness of the trajectory classifier. Using training trials, we first computed a model trajectory for each stimulus. Then, given a test trial's trajectory, we computed, for each moment in time, *t*, a Euclidian distance to the model trajectory of its corresponding true stimulus [*d_T_*(*t*)] (Figure 5A, orange) and a second set of distances to the model trajectories of other stimuli [*d_O_*(*t*)] (Figure 5A, magenta). Finally, these two categories of distances were averaged separately across the set of test trials for a given stimulus (e.g., Figure 5A, top panel, for grating stimuli)(see Materials and Methods). A smaller average distance to the true stimulus compared to other stimuli [*d_T_*(*t*) < *d_O_*(*t*)] implies that patterns appearing around the corresponding moment in time, *t*, are better locked and more specific to the true stimulus and hence carry information about it. After computing these distances, we first expressed the difference between *d_T_* and *d_O_* relative to their trail-to-trial variability as Cohen's *d* (effect size) and then manipulated *τ* (10, 20, 50 and 100 ms). Intuitively, a larger ‘effect size’ indicates that patterns evoked by one stimulus are further apart from patterns evoked by the other stimuli, at a given time point along the trial. The measure has the advantage of being normalized to the variability of *d_T_* and *d_O_* and therefore it can also be interpreted as a signal-to-noise ratio when discriminating among multiple stimuli. We found that for grating stimuli the ‘effect size’ increased monotonically with the increase in the time constant (Figure 5A, bottom), indicating that information was contained predominantly in slow processes (firing rate modulations), in agreement with findings in Figure 3D. In contrast, the time constant optimal for encoding movies dependent on the epoch. The maximum ‘effect size’ was attained on slow timescales (τ = 50–100 ms; Figure 5B) for a movie segment with slow dynamics (slow movement of the entire scene). For a movie segment with faster dynamics the ‘effect size’ was largest sometimes on fast (τ = 10 ms; Figure 5C, blue arrows), intermediate (τ = 50 ms; Figure 5C, yellow arrows), or slow (τ = 100 ms; Figure 5C, red arrows) timescales. These findings were also consistent with results in Figure 3E, where classification performance over the entire duration of the movies indicated that both fast and slow processes were involved in coding. Finally, as would be expected from the excellent classification performance with short time constants when flashed letter sequences were analyzed (Figure 3F), the highest peaks in ‘effect size’ were found for these stimuli when *τ* = 10 and 20 ms (Figure 5D).

## Discussion

### Covering the Spatial and Temporal Aspects of Coding

We have provided a novel methodology able to cope with both the spatial and temporal aspects of coding in a unitary fashion. The spatial aspect is covered by including the simultaneous activity of multiple neurons. Previous limitations of binarization/binning [6]–[9] were overcome by integration with exponentially decaying kernels complemented by a clustering procedure [18] (see also Supporting Discussions in Text S1 for issues related to clustering and further methodological considerations). This allows for the identification of classes of multineuronal activity patterns that evolve on a chosen timescale. Importantly, exponentially decaying functions mimic the shape of post-synaptic currents reasonably well, and hence, the detected patterns resemble input currents received by a potential post-synaptic neuron [15]. Therefore, patterns detected by the method can be considered as instantaneous snapshots of post-synaptic currents converging onto a hypothetical target neuron. In the present study we used the same integration time constant to compute continuous activation traces corresponding to all simultaneously recorded neurons. In the future, more sophisticated strategies can be used if information about the exact synaptic connectivity is known. Since techniques allowing for partial network reconstruction are becoming increasingly available [29]–[31], one might be able to group only pre-synaptic neurons of a given target neuron, and use exponentially decaying kernels having time constants matching the properties of the individual corresponding synapses. This would enable the detailed investigation of the input currents impinging upon a target neuron, i.e. the input current patterns.

### The Collective Behavior of Neurons

Our results are consistent with previous reports emphasizing the importance of collective behavior of neurons. Interactions between multiple cells, mediated by fast synaptic mechanisms, have been found in the medial prefrontal cortex of rats [32]. Population coding has been identified in many structures, including the retina [7], motor cortex [33], and hippocampus [34]. Neuronal ensembles were intensively discussed, from their involvement in chaotic attractors [35] to their role in coding and in the context of neuronal correlations [5], [36]–[39]. Importantly, regardless of the existence or absence of correlation in their firing (dependence or independence), multiple neurons provide a combinatorial code that is more efficient than a population spike count [40]. The analyses provided here extend previous results by detecting and quantifying generalized activity patterns that span an arbitrarily chosen range of timescales. Results indicate that such generalized multi-neuron activation patterns carry much stimulus-related information, allowing a potential classifier to discriminate between different stimulation conditions.

### Relevant Timescales

Neuronal patterns can be defined in many ways, from coincident or delayed spikes, to more general temporal relations between bursts or even between rate fluctuations. Detecting all possible patterns is a hard problem that unfortunately has no general solution. Nevertheless, with the present approach, by using a variety of integration time constants and noting that activation patterns contain the trace of previous spikes, one can also observe more complex relations between spiking of different neurons, e.g. coincident or delayed spikes, bursts or fast rate fluctuations.

A possibility of sorting out relevant relations among spiking events from the vast amount of possible combinations, is to consider that post-synaptic neurons need to integrate their inputs through afferent synaptic currents. These are generated both by fast processes, e.g. supported by AMPA or GABA_A_ transmission (time constants <10 ms [41], [42]) and by slower ones, involving neuromodulators or metabotropic receptors such as GABA_B_ [43] or NMDA (timescales >50–100 ms [44]). In addition, membrane time constants, ranging between 5–30 ms [29], [45], [46], largely determine how the afferent currents influence the dynamics of each cell. Here, we manipulated time constants to study a whole range of integration dynamics, from near coincident (synchronous) spiking (∼1–5 ms) to rate fluctuations (∼50–500 ms) and even mean firing rate (>1 s). In addition, we have used visual stimuli with various temporal properties in order to cover different possible input statistics. Results suggest that the timescale on which informative multineuronal firing patterns evolve depends crucially on the spatiotemporal properties of the stimulus. Similarly, multiple timescales and response dependence on stimulus properties have been recently reported for single neurons in the LGN [13]. Here, however, we focused not only on various types of stimuli and various timescales but also on multineuron firing patterns in the visual cortex, thus extending significantly these previous results. Multineuronal activation patterns containing stimulus specific information evolve on a continuum of different timescales, with time constants ranging from 1–5 ms up to >100 ms. We propose that this flexibility with respect to the use of different timescales allows the visual cortex to represent the spatiotemporal dynamics of stimuli with high fidelity. For ‘slow’ stimuli, slowly depolarized neurons fire stochastically, individual spikes being triggered by temporally dispersed neurotransmitter release [47]. In such cases, fast patterns appear by chance (e.g. on 1–10 ms; Figure 3D, green) and they are locked to stimulus only on a broad temporal scale that reflects the slow modulation by the stimulus. Thus, slow rate modulations trigger a stochastic distribution of faster patterns. In turn, the expression of these fast patterns facilitates the integration of information by post-synaptic neurons having relatively short membrane time constants [5], [48], [49]. For ‘faster’ stimuli (e.g. with abrupt changes in luminance), patterns on short timescales are evoked and they are stimulus-locked with higher precision. The underlying cause is probably the rapid, transient, depolarization. The latter was shown to produce precise spike timing, as is the case for responses to fast flickering [50] or stochastic inputs [51], and to induce precisely spike-synchronized responses, e.g. following saccadic eye movements [52], [53] (see also Supporting Discussions – Slow and Fast Timescales in Text S1).

A fact frequently overlooked is that correlated activity in sensory cortices is ubiquitous because stimuli modulate cortical activity. Current terminology emphasizes the definition of correlation on fast timescales, at the level of individual spikes (i.e. spike-synchrony), but it is important to note that correlated activity could be occurring on various timescales and hence, it is not enough to study correlation only at the level of individual spikes. Especially relevant are the timescales characterizing membrane dynamics and those of synaptic currents whose correlated fluctuations were shown to play a critical role in driving post-synaptic cells [48], [49], [54], [55]. Here we have found that on timescales of 5–20 ms, consistent with the time constants of synaptic currents and neuronal membranes, multineuronal activity patterns carry a large amount of stimulus-related information (Figures 2 and 3). Very high or close to maximum classification performance was attained below a time constant of 20 ms in most cases and for all stimulus types that were investigated here. Importantly, these relatively fast timescales match the temporal learning windows of neurons (<50 ms) [56], and hence, may render spike-timing dependent synaptic plasticity operant for learning informative activation patterns.

### Neuronal Coding: Trajectories in High-Dimensional Spaces?

Dynamic stimuli can evoke informative sequences of events that may be described as trajectories in the neuronal ensemble space, as shown for the olfactory system of honeybees [57], locusts [11], mice [12], and zebrafish [10]. For the visual system, we have shown here that it is also realistic to characterize cortical responses to dynamic stimuli as trajectories in the multidimensional pattern space. This is in contrast to mean spike counts over long temporal windows, which fail to describe such cortical responses appropriately (Figures 3E–F, Figure S3 and Figure S4).

It has been suggested that the brain should be explored from the perspective of a dynamical system evolving in a high-dimensional state-space [11], [12], [36], [57]–[59]. Here, we have shown not only that such an approach can describe well also neuronal responses in the primary visual cortex but were also able to investigate the timescales characterizing these multidimensional trajectories. Importantly, we find that there is no one single relevant timescale along the trajectory but that the coding process may use states expressed on faster or slower timescales depending on the temporal properties of the stimulus (Figure 5).

### Implications for the Perception of Dynamic Visual Scenes

The application of various classifiers described here has revealed that dynamic stimuli evoke informative patterns at particular moments in time. Thus, such dynamic stimuli are best encoded by successions of specific patterns localized at specific moments in time. For different stimuli, similar patterns may appear at different moments in time, yet these stimuli can be properly discriminated if one considers both the identity of the pattern and its temporal occurrence in relation to other patterns. Therefore, it is less likely that a very specific activity pattern can occur exclusively for one stimulus, thereby representing its unique neuronal fingerprint. More likely, dynamic stimuli can only be discriminated if one considers their temporal evolution, i.e. the sequence of activity patterns evoked by the stimuli. These findings imply the existence of some higher-order neuronal mechanisms, able to identify and label different sequences of patterns. Such mechanisms must be able to integrate pattern sequences over long temporal windows, on the order of seconds, but at the moment it is not very clear what these mechanisms might be. Among possibilities, we mention slow synaptic integration based on mGlu/TRPC currents [60], [61] and reentrant connectivity [62]–[64]. The latter can support sustained, reverberating activity that was related previously to working memory [65], [64] but more recently also to linking temporally-delayed events [66]. The road from multineuronal activity patterns to coherent perceptions of dynamic visual scenes probably involves also processes related to visual memory such that timescales operant for stimulus representation (<100 ms) could be bridged with behaviorally relevant timescales (>500 ms).

### Conclusions

Multiple neuron activity carries a large amount of stimulus-related information that is expressed in multineuronal activation patterns. These patterns evolve on multiple timescales, while the timescale that will be expressed for a particular stimulus will depend on the temporal dynamics of the latter. Stimuli with slow dynamics (such as drifting gratings) elicit mostly slow patterns (timescale >20 ms). However, these patterns can be composed of sub-patterns evolving on faster timescales (≤20 ms). The latter are less stimulus-specific and are only weakly stimulus-locked, with a precision comparable to that of the slow timescale (precision >20 ms). Stimuli with fast dynamics, on the other hand, elicit fast patterns precisely time-locked to the stimulus. In all cases patterns evolving on relatively fast timescales (10–20 ms) may be used to represent both slow and fast changing stimuli but the mechanism for the emergence of these patterns may be different and needs to be further investigated. Thus, high-dimensional firing patterns encoding stimulus-specific information are not confined to a single timescale but can span a broad range of timescales, ranging from spike-synchrony to mean firing rate. The dichotomy between spike-synchrony and mean firing rate is therefore artificial and should be avoided, as these two represent only extreme cases of a continuum of timescales that are expressed in cortical dynamics. Timescales consistent with the time constants of neuronal membranes and fast synaptic transmission appear to play a particularly salient role in coding. Finally, cortical responses to dynamic visual stimuli may be described as successions of activity patterns, i.e. trajectories in a multidimensional pattern space, reflecting the temporal characteristics of stimuli. It remains a challenge for future studies to explore systematically both the spatial and temporal aspects of coding and to elucidate how the brain adjusts different timescales in order to faithfully represent the outside world.

## Materials and Methods

### Ethics Statement

Experimental data were recorded from anesthetized and paralyzed adult cats, bred in the facilities of the Max-Planck Institute for Brain Research. All the experiments were conducted in accordance with the European Communities Council Directive of 24 November 1986 (86/609/EEC), according to the guidelines of the Society for Neuroscience and the German law for the protection of animals, overseen by a veterinarian and approved by the local government's ethics committee at Regierungspräsidium Darmstadt with the approval number “Si 1”.

### Experimental Procedures and Recording

Anesthesia was induced with ketamine (Ketanest, Parke-Davis, 10 mg kg^−1^, intramuscular) and xylazine (Rompun, Bayer, 2 mg kg^−1^, intramuscular) and maintained with a mixture of 70% N_2_O and 30% O_2_ supplemented with halothane (0.5%–1.0%). After tracheotomy, the animals were placed in a stereotactic frame. A craniotomy was performed, and the skull was cemented to a metal rod. After completion of all surgical procedures, the ear and eye bars were removed, and the halothane level was reduced to 0.4%–0.6%. After assuring that the level of anesthesia was stable and sufficiently deep to prevent any vegetative reactions to somatic stimulation, the animals were paralyzed with pancuronium bromide (Pancuronium, Organon, 0.15 mg kg^−1^ h^−1^). Glucose and electrolytes were supplemented intravenously and through a gastric catheter. The end-tidal CO_2_ and rectal temperature were kept in the range of 3%–4% and 37°C–38°C, respectively. Stimuli were presented binocularly on a 21 inch computer screen (HITACHI CM813ET) with 100 Hz refresh rate. To obtain binocular fusion, the optical axes of the two eyes were first determined by mapping the borders of the respective receptive fields and then aligned on the computer screen with adjustable prisms placed in front of one eye. The software for visual stimulation was a combination of custom-made programs and a stimulation tool, ActiveSTIM (www.ActiveSTIM.com). Data were recorded from area 17 of 6 adult cats by inserting multiple silicon-based multi-electrode probes (16 channels per electrode) from the Center for Neural Communication Technology at the University of Michigan (Michigan probes). Each probe consisted of four 3 mm long shanks that were separated by 200 µm and contained four electrode contacts each (1,250 µm^2^ area, 0.3–0.5 MΩ impedance at 1,000 Hz, inter-contact distance 200 µm). Signals were amplified 10,000× and filtered between 500 Hz and 3.5 kHz and between 1 and 100 Hz for extracting multi-unit (MU) activity and local-field potentials (LFP), respectively. The waveforms of detected spikes were recorded for a duration of 1.2 ms, which allowed the later application of offline spike-sorting techniques to extract single units (SU). For spike-sorting we have used a custom made software that first computed principal components of spike waveforms (for each channel independently) and then applied clustering to group waveforms of similar shapes that were further assumed to be generated by the same neuron.

### Datasets

The investigated neuronal activity was acquired in response to a variety of visual stimuli. Recordings from 6 different cats are below coded with dataset names to facilitate easy identification. The dataset naming conventions are: catID-sessionID, e.g. col05-e08 (cat col05, session e08). In all datasets, stimuli were presented in a randomized order.

#### Datasets with drifting sinusoidal grating stimuli (col05-e08a, col05-e08b, col05-e06, col07-g01, and col08-e19)

Sinusoidal gratings moving in 12 directions in steps of 30° were presented in trials of 4,800 ms duration (1,000 ms spontaneous activity, 3,500 ms stimulus, 300 ms OFF-response). Gratings spanned 12° of visual angle, had a spatial frequency of 2.4° per grating cycle and were presented at a speed of 2° per second. Stimuli were presented 20 times each. The analyses were conducted in three different cats (col05-, col07-, and col08-), on a total of 5 datasets that were spike-sorted and yielded different numbers of SUs with overlapping receptive fields. **Cat 1 (col05)**: dataset col05-e06 consisted of 46 SUs and was used in Figure S3A. Dataset col05-e08a consisted of 26 SUs and was used in Figure 1B, Figure 2A, Figure 3A and 3D, Figure 4A, Figure 5A, Figure S1, Figure S2, and Figure S5. Dataset col05-e08b is the same as col05-e08a, except that it was re-sorted using another criterion that yielded 47 SUs; it was used in Figure S3B. **Cat 2 (col07)**: dataset col07-g01 consisted of 32 SUs and was used in Figure S3C. **Cat 3 (col08)**: dataset col08-e19 consisted of 26 SUs and was used in Figure S3D. Datasets with 12 directions of drifting gratings were used in several previous studies to determine the direction preferences of neurons [67], the oscillation frequencies of responses to different orientation preferences [68] or to investigate the entropy and network topology of synchronized responses [9].

#### Dataset with natural stimuli (cer01-a50)

Three movies with natural images were presented to the cat (one recorded by the authors, two extracted from “The Greatest Places” movie provided by the Science Museum of Minnesota). The movies contained indoor and outdoor scenes with various image statistics (slow moving, fast moving, dark, light, etc) and had a resolution of 800×600 pixels, spanning the entire screen. Each movie was 28 seconds long and was presented 20 times. Analyses were performed on 22 simultaneously recorded SUs from **Cat 4 (cer01)**. The dataset was used in Figure 3B and 3E, Figure 4B and 4C, Figure 5B and 5C, Figure S1, Figure S2, and Figure S5. The dataset with natural stimuli was previously used when the visualization technique based on 3D Kohonen maps was introduced [18].

#### Dataset with flashed graphemes (col10-d24a)

Stimuli consisted of 26 letters (A–Z), 8 digits (0–7), 3 small size letters (A–C), 3 large size letters (A–C) and 9 rotated letters (A–C, rotated at 90°, 180° and 270°). Each grapheme was white on a black background, spanning approximately 5°–7° of visual angle. Trials were 1,200 ms long with stimuli flashed for 100 ms (between 500 ms and 600 ms). Each stimulus was presented 50 times. Analyses were performed on 20 SUs recorded from **Cat 5** (**col10**) and are presented in Figure 2B.

#### Datasets with flashed letter sequences (col10-d24b, cer01-a47, col13-a20)

Stimuli consisted of three (“A-B-C”, “A-D-C”, “D-B-C” for col10-d24b) or four letter sequences (“A-B-E”, “A,-D-E”, “C-B-E”, “C-D-E” for cer01-a47 and col13-a20). Each letter was flashed for 100 ms, with an inter-letter-interval of 100 ms. Trials were 1,200 ms long with stimuli presented at 500, 700 and 900 ms for three letter sequences (col10-d24b) and 3,800 ms long with stimuli presented at 500, 700, 900 and 1,100 ms for four letter sequences (cer01-a47 and col13-a20). These stimuli typically entrain rhythmic changes [69] in firing rates as a result of alternating on- and off-responses to the letters appearing along the presentation sequence (see Figures 1B, 3, 4 and S7 in Nikolić et al. 2009 [15]). Stimuli were presented 50 (col10-d24b) or 300 (cer01-a47 and col13-a20) times each. Analyses were performed on three different cats. **Cat 5 (col10)**: dataset col10-d24b consisted of 20 SUs and was used in Figure 3C and 3F, Figure 4D, Figure 5D, Figure S1 and Figure S2. **Cat 4 (cer01)**: dataset cer01-a47 consisted of 45 SUs and was used in Figure S4A. **Cat 6 (col13)**: dataset col13-a20 consisted of 45 SUs and was used in Figure S4B.

Datasets with flashed graphemes and flashed letter sequences were previously used to probe the availability of stimulus related information in neuronal responses over time [15].

### Low-pass Filtering of Spikes and Definition of Activity Vectors

Spike trains were low-pass filtered using an exponentially decaying kernel, using the same procedure presented elsewhere [18]. For each neuron *i*, a continuous signal, called *activation*, *a_i_*(*t*) was obtained using the formula:
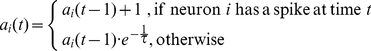
(1)where, *a_i_*(*t*) is the activation corresponding to neuron *i* at time *t*, *τ* is the decay (integration) time constant.

We defined an activity vector at time *t* as:
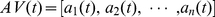
(2)where, *n* is the number of analyzed neurons.

### Kohonen Mapping

The activity vectors of each recording session were clustered and mapped onto a 3D space using 3D Kohonen maps (3DKM), to also enable the visualization of patterns [18]. The extension of 3DKM over the classical 2D Kohonen map consists in using a *N*×*N*×*N* lattice, instead of *N*×*N*. Each map element contained a vector of dimension equal to the dimensionality of the input space, termed *model vector*. At each step *k* of the learning algorithm, the 3DKM learned an activity vector (*AV_k_*) by finding its most similar model vector in the map (best-matching units – BMU) and altering it and its neighbors [23]. The amount of change and the radius of the neighborhood are given by two monotonically decreasing functions: *L*(*k*) and *R*(*k*), respectively:
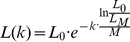
(3)where, *L*(*k*) is the learning rate, modulating how much model vectors were changed at training step *k*. *L_0_* and *L_M_* are initial and final learning rates. We used *L_0_* = 1 and *L_M_* = 0.01. The total number of training steps is denoted by *M*.
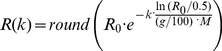
(4)where, *round* denotes the rounding to the nearest integer, *R*(*k*) specifies the neighborhood size around the BMU within which elements were allowed to learn at step *k. R_0_* is the initial radius of the neighborhood. *g* is the percentage of *M* after which *R* becomes 0 (only the BMU is modified for *R* = 0). We used *R_0_* = *N*/2 and *g* = 66 (66% of steps were used to establish the topology of the map and the last 34% of the steps to fine-tune the representation of activity vectors in the map).

Within the learning neighborhood model vectors further away from the BMU change less than the ones closer to it, by multiplying the learning rate with a 3D Gaussian envelope having a standard deviation of *R*(*k*)/3:
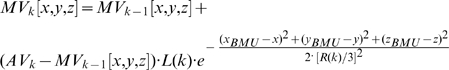
(5)where, *MV_k_*[*x*,*y*,*z*] is a model vector, at step *k* of the training, located within the neighborhood of the BMU (distance from BMU ≤*R*(*k*)) at position (*x*,*y*,*z*) in the 3D lattice. (*x*_BMU_, *y*_BMU_, *z*_BMU_) is the position of the BMU in the 3D lattice. *AV_k_* is the activity vector that is learned at step *k*, *L*(*k*) and *R*(*k*) are, respectively, the learning rate and the size of the neighborhood at step *k*.

For details regarding convergence and stopping criteria see also our previous report [18]. Here, we used maps that included 1,000 points (clusters) in the 3D lattice (*N* = 10). Thus, for *N* = 10 there were 1,000 patterns available to describe a dataset.

### Pattern Specificity

The specificity *SP_p_*(*j*) of a pattern *p* (model vector *MVp*) to a given stimulus *j* was computed as:
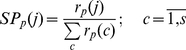
(6)where, *r_p_*(*j*) is the number of occurrences of pattern *p* in all trials belonging to stimulus *j*, *r_p_*(*c*) is the number of occurrences of pattern *p* in all trials belonging to stimulus *c*, and *s* is the number of stimuli.

Pattern specificity is thus a function of a stimulus set, and has a value for each stimulus, between 0 (never occurs for that stimulus) and 1 (occurs only for that stimulus). The sum of specificities of a pattern across the stimulus set always amounts to 1. See also the same concept, termed *Pattern Specificity Index*, explained in [18].

### Classifiers

Datasets were first half-split by randomly choosing half the trials for the training set and half for the testing set, for each stimulus condition. After training and classification, performance was computed as a ratio between the number of correctly classified trials and the total number of trials that were classified. The half-splitting procedure was repeated 1,000 times to compute the mean and standard deviation of classification performances.

#### Mean rate classifier

For the training set, model firing rate vectors were computed for each stimulus, as follows:
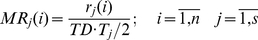
(7)where, *MR_j_*(*i*) is the entry corresponding to neuron *i* in the model rate vector for stimulus *j*, *r_j_*(*i*) is the spike count of neuron *i* for all training trials corresponding to stimulus *j* (*T_j_*/2 training trials), *TD* is the duration of a trial in seconds, *T_j_* is the total number of trials recorded for stimulus *j*, *n* is the number of neurons and *s* is the number of stimuli.

For a trial *l*, from the testing set, a mean firing rate vector (*RV_l_*) was first computed:
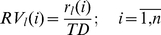
(8)where, *RV_l_*(*i*) is the entry corresponding to neuron *i* in the mean rate vector for trial *l*.

Finally, the trial *l* to be classified was assigned to a stimulus condition *SC_l_* by finding the closest model rate vector (*MR_j_*) in Euclidean distance:
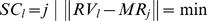
(9)


The mean rate classifier is similar to a Maximum Likelihood classifier. See also Classifiers Explained Intuitively in Text S1.

#### Specificity classifier

This classifier takes into account the specificity of patterns appearing in a test trial *l* and builds specificity scores corresponding to each stimulus condition *j*:
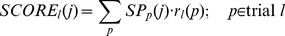
(10)where, *SCORE_l_*(*j*) is the score corresponding to stimulus *j*, computed for trial *l*, and *SP_p_*(*j*) is the specificity of pattern *p* for stimulus *j* computed only on the train trials, *r_l_*(*p*) is the number of occurrences of pattern *p* in trial *l*, and *p* spans all patterns expressed in trial *l*.

The test trial *l* is assigned to stimulus condition *SC_l_* that has the highest corresponding specificity score:
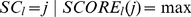
(11)


Since this classifier considers all patterns in a trial and disregards their position in the trial, it does not take into account the dynamics of the cortex in response to the dynamics of the stimulus and hence it does not consider stimulus-locking. The specificity classifier is similar, although not equivalent, to a Naïve Bayesian classifier in that it classifies a trial based on probabilities that a pattern is evoked by a given stimulus (pattern specificities). However, unlike the Bayesian classifier, we use a sum of weighted probabilities (specificities) to avoid the problems caused when a pattern is not expressed at all in a given condition (the Bayesian product of probabilities would be zero). See also Classifiers Explained Intuitively in Text S1.

#### Trajectory classifier

The trajectory in the multidimensional pattern space was defined by first segmenting each trial into non-overlapping windows of size equal to the integration time constant (*τ*) that was used to compute the patterns. After segmentation of trial *l*, an average pattern *MVA_l_*(*w*) was computed for each window *w* of size *τ* bins, by taking the average of patterns *p* appearing in the respective window:
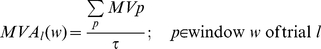
(12)where, *MVp* is the model vector of pattern *p*.

A trajectory of a trial *l* was thus represented as a sequence of average model vectors [*MVA_l_*(1), *MVA_l_*(2), …, *MVA_l_*(*Nw*)], where *Nw* is the total number of windows resulted after the segmentation of the trial. Using the training trials, a set of model trajectories (*MT_j_*) was computed for each stimulus *j*, by averaging trajectories corresponding to training trials that belong to the same stimulus:
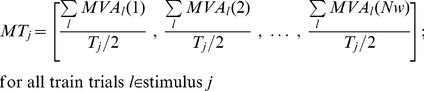
(13)where, *MT_j_* is the model trajectory for stimulus *j*, *MVA_l_*(*w*) are the average model vectors corresponding each window *w*, *T_j_* is the total number of trials recorded for stimulus *j*. Sums run over the *T_j_*/2 trials belonging to the training set for stimulus *j*.

For a new test trial *l* to be classified, the distance *DT_l_*(*j*) between the trajectory of the trial and each model trajectory *j* was computed by summing up Euclidean distances for all windows *w*:
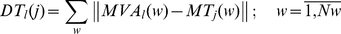
(14)where, *DT_l_*(*j*) is the distance between the trajectory corresponding to trial *l* and the model trajectory corresponding to stimulus *j*, *MVA_l_*(*w*) are the average model vectors corresponding each window *w*, and *Nw* is the total number of windows resulted after the segmentation of the trial.

The test trial *l* is assigned to stimulus condition *SC_l_* that yields the lowest distance between its corresponding model trajectory and the trajectory of the trial:
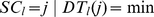
(15)


The trajectory classifier is also a Maximum Likelihood classifier. It accumulates point by point distances between the test trajectory and model trajectories (computed as averages for each stimulus) to estimate the stimulus inducing the most similar temporal structure to the test trial. See also Classifiers Explained Intuitively in Text S1.

### Time Resolved Distances and ‘Effect Size’ for Trajectories in the Pattern Space

To identify where informative patterns were located in time, we computed, for trajectory classifiers, two time resolved distances, as follows: For all test trials *l* of a given stimulus *ts*, we computed the average time-resolved distance to the model of that stimulus (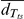) and the average time-resolved distance to the models of other stimuli (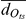):
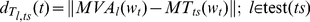
(16)

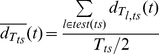
(17)

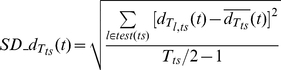
(18)


where, 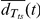
is the average distance, from the trajectory 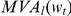 of a test trial, belonging to stimulus *ts*, to the model trajectory 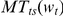 of stimulus *ts* at time *t* (window *w_t_* centered at time *t*); 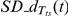 is the standard deviation of 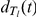 over test trials 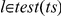; *test*(*ts*) is the set of test trials for stimulus *ts*; *T_ts_* is the number of trials recorded for stimulus *ts*.
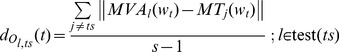
(19)

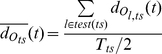
(20)

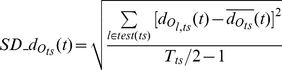
(21)where, 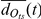
is the average distance, from the trajectory 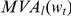 of a test trial, belonging to stimulus *ts*, to the model trajectories 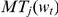 of other stimuli *j≠ ts*, at time *t* (window *w_t_* centered at time *t*); 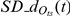 is the standard deviation of 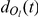 over test trials 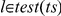; *test*(*ts*) is the set of test trials for stimulus *ts*; *T_ts_* is the number of trials recorded for stimulus *ts*; *s* is the total number of stimuli.

The two time-resolved distances 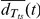
and 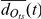 show, for each instant in time, how close is on average, a trajectory corresponding to a test trial of a stimulus *ts* to its true model trajectory, and to the model trajectories of other stimuli, respectively. At moments *t* in time where 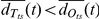, the trajectory of the test trial is closer to the model of its corresponding true stimulus *ts* than to models of other stimuli, and, hence, it contains specific information about stimulus *ts*. As compared to the trajectory classifier described in Equations 12–15, here the window *w* was always slid with at most a 5 ms step such as to yield a good temporal resolution in identifying zones with high information content. Note that classification performance plots (Figure 3, Figure S3 and Figure S4) remain unchanged even if windows are overlapping and slid with a 5 ms resolution.

To quantify how much closer is a test trial to the model of its true stimulus compared to the models of other stimuli, we computed a time-resolved measure of *Cohen*'*s d* ‘effect size’ by considering the two time-resolved distances mentioned above:
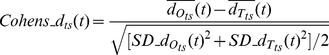
(22)


A large, positive value of *Cohens_d_ts_*(*t*) for a given moment *t* in time means that the trajectory of a test trial belonging to stimulus *ts* is reliably closer to the model of its true stimulus as compared to the models of other stimuli, for that moment in time *t*. The effect size is normalized with respect to the standard deviations of distances. Therefore it represents not only how much closer are patterns, around time *t*, on average to the patterns of the true stimulus, but also how closer they are with respect to the variability across trials. This ‘effect size’ measure can also be interpreted as the variability-normalized width of the separatrix between different pattern classes in multidimensional space.

## Supporting Information

Text S1 Supporting Information text.
(PDF)

Figure S1 Classification performance of the trajectory classifier applied on data clustered with 3D Kohonen maps (red) and on unclustered data (blue). Error bars represent s.d.
(TIF)

Figure S2 Classification performance of the specificity and trajectory classifiers applied on data clustered with 3D Kohonen maps (red) and with K-Means (blue). Error bars represent s.d.
(TIF)

Figure S3 Reproduction of classification results for datasets evoked by drifting sinusoidal gratings. (A) and (B), Classification results for two datasets recorded from the same cat as in Figure 3D. The example in (B) is the same dataset as in Figure 3D but resorted according to different criteria. (C) and (D), Reproduction of classification results in two additional cats. Error bars represent s.d.
(TIF)

Figure S4 Reproduction of classification results from Figure 3F. (A) and (B), Results on datasets recorded in response to flashed letter sequences, from two additional cats. Error bars represent s.d.
(TIF)

Figure S5 Effect of jitter on datasets with slow stimuli after bursts have been eliminated by keeping only the first spike in each burst. Error bars represent s.d. over independent jitters.
(TIF)
