# Supplementary material for: Timescales of Multineuronal Activity Patterns Reflect Temporal Structure of Visual Stimuli
Source: PLoS One. 2011 Feb 8;6(2):e16758. doi: 10.1371/journal.pone.0016758 (PMC3035626; doi:10.1371/journal.pone.0016758)
Supplement: Text S1 — Supporting Information text. (PDF) [file pone.0016758.s001.pdf]

Supporting Information for

Timescales of Multineuronal Activity Patterns Reflect Temporal Structure of Visual Stimuli

Ovidiu F. Jurjuț, Danko Nikolić, Wolf Singer, Shan Yu, Martha N. Havenith, Raul C. Mureşan*

* To whom correspondence should be addressed. E-mail: muresan@coneural.org

The Supporting Information includes:

1. Considerations Regarding Clustering
2. Classification
    - Classifiers Explained Intuitively
    - Consistency of Classification Results
3. Supporting Discussions
    - Coping with Multidimensional Neuronal Patterns
    - Methodological Considerations
    - Slow and Fast Timescales
4. Supporting References

1. Considerations Regarding Clustering

Is clustering necessary? The mean rate classifier is applied directly on the spike-trains and therefore is not affected by clustering. The trajectory classifier can also operate directly on the unclustered activity vectors because it relies just on Euclidean distances. However, the

specificity classifier is dependent on clustering because it needs a finite set of patterns on which to compute statistics such as specificity and frequency of occurrence. Activity vectors cannot be directly used in this latter case and clustering must be performed to identify model vectors, because of the following reasons. Our approach (and others as well – see Baker and Gerstein [1]) does not binarize and bin multineuronal spike data, as is the case in other methods [2, 3] where a finite set of 2^n vectors (n is the number of neurons) is obtained. By convolving spike trains with exponentially-decaying kernels and then sampling the resulting traces we obtain activity vectors with real-valued elements. As a consequence, activity vectors can represent an infinity of possible combinations of values and therefore there is virtually an infinite possible number of activity vectors. Two activity vectors will be considered mathematically different even if the difference is in a single element and it is infinitesimally small. Clustering solves this problem and yields a finite set of representative model vectors (patterns), albeit with the tradeoff of an approximation error. The specificity classifier can then operate on this finite set of representative patterns and can compute pattern-related statistics (stimulus specificity, frequency of occurrence, and so on).

To be able to compare results across the specificity and trajectory classifiers, data were clustered first and only then used in classification for both these classifiers (both had the same input). Nevertheless, to test whether results could be different if we didn’t cluster data first, we reclassified data with the trajectory classifier by feeding it directly with activity vectors. We found that classification performance and its dependence on integration constant were

very similar (almost identical) with the case where data was first clustered (Figure S1). This indicates that clustering does not affect results and does not lead to any spurious conclusions.

Kohonen versus K-Means clustering. To verify that results were not a byproduct of clustering with Kohonen maps, we repeated the analysis in Figures 3D-F using K-Means clustering. For each integration time constant, we ran the classical K-Means algorithm 10 times (with k=1000, to match the number of nodes in the Kohonen map) and applied the specificity and trajectory classifiers selecting the run that yielded the highest classification performance. In Figure S2 we compare the classification performance curves from Figures 3D-F, which were obtained with 3D Kohonen clustering, with those obtained with K-Means clustering. In all cases curves were very similar, almost overlapping. Sometimes K-Means enabled slightly higher classification performance even if, in general, it had higher approximation error. Nevertheless, these differences were very small. Similar results were obtained also with Linde-Buzo-Gray clustering algorithm [4]. Thus, the particular clustering algorithm is of little importance to the results reported here.

2. Classification

Classifiers Explained Intuitively

Classification is a crucial point for the message of the present study. Therefore, understanding how the classifiers were constructed and what conclusions may be drawn from their performance is essential. Classification always involves two important concepts:

input features and output classes. Input features represent the features of the data that are extracted and on which the classifier relies on in order to classify. For the three types of classifiers presented here, the input features were: mean firing rate vectors for the mean rate classifier, specificity of patterns for the specificity classifier, and average patterns in successive time windows for the trajectory classifier. The output classes were always represented by the identity of stimuli for a particular dataset: the direction of the drifting grating for sinusoidal grating datasets, the movie that was presented for natural movie stimuli, and the sequence of letters that was shown to the cat for the flashed letter sequences datasets. The classifiers find a correspondence (mapping function) between the input features and the output classes. How good this correspondence is, reflects to a large degree the informative value of the feature that the classifier relies on.

After choosing the classifier, classification involves two distinct steps: training and testing. Training involves finding the mapping function from input features to output classes by 'learning', for each class, either a function or a model of the input. Testing consists in identifying, for a new sample that was not used in training, the class it belongs to. Here, half the data (half of the trials) were used for training classifiers and half for testing. To eliminate any sampling bias, classification was repeated 1,000 times, and for every run trials were randomly half-split into disjoint train and test sets. Classification performance is measured in percent correct identifications of the class for the test set. Unlike statistical methods (e.g. effect size or significance), in which two different conditions are compared, classification

performance reflects how well, on average, a stimulus can be discriminated from all the other stimuli used in recording a particular dataset.

The mean rate classifier. During training, an average rate vector, called model rate vector, is built as a model for each stimulus (see main text Eq. 7). During testing, when a new trial is presented to the classifier, a rate vector is first computed on the spike-trains corresponding to the trial (see main text Eq. 8). This rate vector is then compared to model rate vectors corresponding to all stimuli. The closest model vector is considered as a match and its stimulus is assigned as the identified stimulus for the trial (see main text Eq. 9). Classification performance is computed as the number of correct identifications divided by the total number of test trials. It reflects how well (fraction or percentage of cases) a stimulus can be discriminated from all the others, based on the mean firing rate.

The specificity classifier. During training, the specificity of all patterns is computed for each stimulus. Only trials in the training set are used. Specificity of a pattern is a function of stimulus and takes values between 0 (not specific) and 1 (pattern only appeared for a given stimulus; see main text Materials and Methods). It reflects the estimated probability of the pattern to appear for a given stimulus across the set of stimuli. After training, a pattern has a given specificity assigned for each stimulus. Specificity of the pattern to a stimulus is computed simply as the number of cases in which the pattern appears for the stimulus divided by the total number of appearances of the pattern in the training set. As a consequence, specificity does not contain any information about the location of the pattern in

the trial (moment in time where the pattern appears). Therefore, the stimulus-locking of patterns is completely ignored. During testing, for a new test trial to be classified, a scoring vector is built that contains a score corresponding to each stimulus. For each pattern appearing in the trial, the specificity vector of the pattern (vector containing the specificities of the pattern to all stimuli) is added to the scoring vector (see main text Eq. 10). After all the patterns in the trial have been processed, the classification decision is reached: the stimulus with the highest corresponding value in the scoring vector is assigned to the test trial (see main text Eq. 11). Thus, this classifier's performance reflects how specific to the true stimulus are the patterns appearing in the entire trial.

A problem arises with both the specificity and mean rate classifiers. Different, dynamic, spatio-temporal stimuli may elicit similar patterns at different moments in time. Because for dynamic visual stimuli the stimulus identity should be defined as a temporal sequence of spatial patterns that translate into a temporal sequence of spatial activation patterns in the cortex, it is unfair to compare patterns that appear at different moments in time. This means that the specificity of a given pattern (or the average model rate vector) for a given stimulus will be negatively affected if the same pattern appears in a different context (location in the trial) for another stimulus. Consider the following example: two distinct movies with natural scenes are presented to the cat. For the first movie, an edge with a given orientation passes neuronal RFs at moment 300 ms. For the second movie, a similar edge passes the RFs at moment 1,300 ms. The specificity of the pattern that is evoked by this edge will be small

because the pattern appears equally for both stimulus conditions. However, taking into account that these are dynamic stimuli, a better way to describe the stimulus is to say that the edge crosses the RFs at a specific moment in time. For this reason, averaging over the entire trial (mean rate) or computing pattern specificity cannot provide a good account of the dynamic coding process that takes place. The temporal component (stimulus locking) needs to be also considered.

The specificity and mean rate classifiers will provide a good classification performance only if the response to each stimulus in the set is independent (the patterns for a given stimulus are not shared with other stimuli), or there is a statistical difference in the average drive that neurons receive for different stimuli from a particular set. When these conditions cannot be achieved (which is usually the case for dynamic stimuli) both classifiers perform rather poorly (Figure 3F). To solve these problems we introduced the trajectory classifier.

The trajectory classifier. During training, a model trajectory is computed for a stimulus, as a sequence of average patterns appearing at successive moments in time (see main text Eq. 13). During testing, a trajectory corresponding to a trial (sequence of patterns) is compared point-by-point to all model trajectories (see main text Eq. 14). The stimulus corresponding to the closest trajectory is assigned to the test trial (see main text Eq. 15). This classifier is intuitively better than the other two because it is general enough to describe the cortical response to both dynamic and static stimuli. It considers that a temporal succession of spatial input patterns is reflected in a temporal succession of multi-neuronal activation patterns.

Importantly, the size of the window in which patterns are averaged (in order to compute a given point of the trajectory) was always taken equal to the timescale of the patterns, τ (see main text Materials and Methods). Therefore, when a given timescale was chosen, the classification performance of this classifier reflected the precision with which patterns that evolve on timescale τ were locked to the stimulus in a window of the same size τ.

Relation between classifiers. By manipulating the timescale (integration time constant) of patterns and observing the behavior of the different classifiers one has the possibility to investigate how the coding of a stimulus comes about. Note that the mean rate classifier is obviously not influenced by the timescale as it is not relying on patterns but on spike-counts. It rather represents an upper limit timescale (equal to the length of the trial), and as a consequence the other two classifiers' performance approaches that of the mean rate as the timescale increases towards the trial length (Figure 3D-F). Because the mean rate only reflects if, on average, there is a stimulus specific bias in the spike-count vectors, we will next discuss only the relation between the specificity and trajectory classifiers for different timescales.

Case 1: High performance of the specificity classifier but low performance of the trajectory classifier for fast timescales (1-5 ms; Figure 3D, Figure S3). In this case, the high performance of the specificity classifier indicates that fast patterns are stimulus specific. However, the low performance of the trajectory classifier shows that these stimulus specific patterns are not locked to the stimulus with a precision comparable to the timescale (τ). Such fast patterns might arise from chance synchronization of neurons, with a precision of τ, induced by slower

rate modulations. Since in Figure 3D the trajectory classifier eventually catches up with the specificity classifier for larger time constants (> 20 ms), it can be concluded that slower coding processes predominate and locking occurs with a precision broader than 20 ms.

Case 2: The pattern trajectory has a high performance for both small and large timescales. This means that there are both fast patterns, precisely stimulus-locked, and slow patterns, stimulus-locked on a slower timescale (Figure 3E).

Case 3: Low performance of specificity classifier but a high peak performance of the trajectory classifier. This is an indication that the stimulus evokes dynamical responses over the trial and that different stimuli might elicit similar patterns at different moments in time. The timescale at which the pattern trajectory attains its peak performance represents the average timescale on which informative patterns evolve and the precision of their locking to the stimulus (Figure 3F, Figure S4).

Consistency of Classification Results

To check the consistency of classification results, we tested whether results could be reproduced in two additional animals. Datasets were available only for drifting sinusoidal gratings and for flashed letter sequences. For drifting sinusoidal gratings, we first considered two additional datasets (col05-e06: Figure S3A, col05-e08b: Figure S3B) recorded from the same cat (**col05**) as the one from Figure 3D. The first dataset (Figure S3A) provided similar results as in Figure 3D. The second dataset (col05-e08b) corresponded to the same session as the one from Figure 3D (col05-e08a) but it was spike-sorted with different criteria. After the

spike-sorting clustering procedure separated units into many groups of waveforms, our spike-sorter allowed for a flexible choice of manually merging waveform groups to form units. In this second dataset, we decided to apply less merging and thus to include more single-units (a more aggressive separation into single-units). Results shown in Figure S3B reveal improvement in classification performance for all classifiers when more units were included, as compared to Figure 3D. In Figure S3C and S3D results are depicted for two additional cats (**col07** and **col08**). The relation between classifiers remained the same for small timescales (larger performance for specificity classifier than for trajectory classifier) and consistent with the conclusions in the main text. Furthermore, qualitatively the performance curves look remarkably similar regardless of the number of electrodes used or the particular animal that was included in the analysis. Also, spike-sorting does not seem to influence the relation between different classifiers across the spectrum of timescales that were investigated.

Results corresponding to the dataset with flashed letter sequences (Figure 3F) were also reproduced in two additional cats (**cer01** and **col13**; Figure S4). In all cases, the largest performance was attained by the trajectory classifier and for small timescales of 5 or 10 ms.

3. Supporting Discussions

Coping with Multidimensional Neuronal Patterns

We have applied Kohonen clustering in addition to low-pass filtering spikes with exponentially decaying kernels. Mapping and clustering have been frequently used as

important tools in characterizing other multidimensional systems [5-8]. However, to the best of our knowledge, these methods have not been exploited before to their full potential in exploring neuronal coding.

The clustering we used could have a two-fold effect, both with a negative and beneficial impact. Because activity vectors that occur consistently will find a robust representation in the clusters (model vectors), some very rarely appearing ones could be missed. However, the same logic renders clustering efficient in emphasizing only effects that are really salient, i.e. patterns that occur robustly. In addition, since we minimized the approximation error for clustering, model vectors in the map resemble very closely the actual appearing patterns and one can always trace back, for a given pattern, the spike constellation that evoked it at any given moment in time (e.g., see Figure 2B). Thus, clustering enables an automated and precise discovery of robustly occurring classes of spiking patterns.

Methodological Considerations

In the process of computing patterns we have employed two procedures: low-pass filtering and Kohonen clustering. While the first one could claim a direct biological correspondence (synaptic integration), the biological relevance of the second is less obvious. Clustering can in fact be understood in a biological context since it involves only averaging and competition. Patterns can be preferentially learned through Hebbian rules by the synapses of competing neurons, and such models are described frequently to account for self-organized learning [9-13]. Using a representative model vector for a given activity vector can

be linked to a clustering of the input space: When new stimuli are presented, neurons with the closest synaptic structure to input patterns will be activated preferentially, in a manner similar to our labeling of activity vectors by model vectors.

The classifiers we have introduced here are simple and involve only few assumptions, computing just averages and Euclidean distances. In the brain, average activation patterns could be computed by activity-dependent synaptic potentiation and depression [14]. Computing distances is somewhat equivalent to competition and selection, where, for example, only the neuron with the closest synaptic pattern will be activated [11] or it will be activated earlier [9, 12], subsequently inhibiting competing representations. Due to their simplicity, classifiers described here allow for direct and meaningful conclusions about the biological importance of the extracted features: mean firing rates, pattern specificity, or trajectories in the multidimensional space.

Slow and Fast Timescales

For the case of stimuli with slow dynamics (e.g. low spatial frequency gratings), because of the predominantly limited integration window of neurons (< 30 ms), correlated activity on the slow timescales (> 100 ms) must somehow be reflected on the fast timescale as well in order to be detected. This process can be implemented either through chance synchronization of neurons on faster timescales (10-20 ms), as neurons stochastically discharge with a probability modulated by the slow process, or through more coordinated firing that is internally regulated by the network [15]. We found that, for drifting gratings, specific patterns

on fast timescales (1-20 ms) appear in a fashion modulated by the slow drifting grating and allow for excellent discrimination of stimuli (Figure 3D and Figure S3, green). These patterns were stimulus locked with a precision > 20 ms. Thus, slow processes can be represented by patterns evolving on faster timescales that are consistent with the biological time constants of neurons and synapses. For the case of stimuli with faster dynamics, neurons have a tendency to fire more precisely [16] and faster patterns, more precisely locked to the stimulus, may be involved in coding (Figure 3F). In such cases coding may be biased towards more synchronous discharges on timescales of 5-10 ms.

Correlations induced by fast dynamic stimuli may be represented naturally on the fast timescales compatible with the time constants of neuronal membranes and AMPA neurotransmission. They arise because of the reliable spiking of neurons when these are depolarized by currents with fast dynamics [16]. On the other hand, correlations induced by slower stimuli need to be converted to correlations on the fast timescale in order to be effective in driving post-synaptic neurons [17-19]. Because in response to slow inputs neurons tend to fire more randomly [16], faster correlations may naturally be induced by chance, from slower firing probability modulations. In addition, other processes, such as coordination of neuronal assemblies or fast oscillations may also be involved in recoding the slow correlations of the stimulus into faster correlations efficient for driving other post-synaptic neurons [15]. In any case, the characteristic timescale of neuronal membranes and synaptic currents seems to play a crucial role in how the timescale of the stimulus is coded into

neuronal firing patterns because the performance of both pattern classifiers (specificity and trajectory) peaked already at timescales around 10-20 ms.

4. Supporting References

1. Baker SN, Gerstein GL (2000) Improvements to the sensitivity of gravitational clustering for multiple neuron recordings. Neural Comput 12: 2597–2620.
2. Schneidman E, Berry MJ, Segev R, Bialek W (2006) Weak pairwise correlations imply strongly correlated network states in a neural population. Nature 440: 1007–1012.
3. Grün S, Diesmann M, Aertsen A (2002) Unitary events in multiple single-neuron spiking activity: I. Detection and significance. Neural Comput 14: 43–80.
4. Linde Y, Buzo A, Gray RM (1980) An algorithm for vector quantizer design. IEEE transactions on communications systems COM-28: 84-95.
5. Frey BJ, Dueck D (2007) Clustering by passing messages between data points. Science 315: 972–976.
6. Kohonen T (2001) Self-Organizing Maps. Springer series in information sciences. Berlin, Springer-Verlag, 3 edition.
7. Roweis ST, Saul LK (2000) Nonlinear dimensionality reduction by locally linear embedding. Science 290: 2323–2326.
8. Tenenbaum JB, de Silva V, Langford JC (2000) A global geometric framework for nonlinear dimensionality reduction. Science 290: 2319–2323.

9. Delorme A, Perrinet L, Thorpe SJ (2001) Networks of integrate-and-fire neurons using Rank Order Coding B: Spike timing dependent plasticity and emergence of orientation selectivity. Neurocomputing 38-40: 539 - 545.

10. Lazăr A, Pipa G, Triesch J (2007) Fading memory and time series prediction in recurrent networks with different forms of plasticity. Neural Netw 20: 312–322.

11. Lücke J, von der Malsburg C (2004) Rapid processing and unsupervised learning in a model of the cortical macrocolumn. Neural Comput 16: 501–533.

12. Masquelier T, Thorpe SJ (2007) Unsupervised learning of visual features through spike timing dependent plasticity. PLoS Comput Biol 3: e31.

13. Triesch J (2007) Synergies between intrinsic and synaptic plasticity mechanisms. Neural Comput 19: 885–909.

14. Bi G, Poo M (2001) Synaptic modification by correlated activity: Hebb's postulate revisited. Annu Rev Neurosci 24: 139–166.

15. Singer W (1999) Neuronal synchrony: a versatile code for the definition of relations? Neuron 24: 49–65, 111-25.

16. Goaillard JM, Marder E (2006) Dynamic clamp analyses of cardiac, endocrine, and neural function. Physiology (Bethesda) 21: 197–207.

17. Bruno RM, Sakmann B (2006) Cortex is driven by weak but synchronously active thalamocortical synapses. Science 312: 1622–1627.

18. König P, Engel AK, Singer W (1996) Integrator or coincidence detector? The role of the cortical neuron revisited. Trends Neurosci 19: 130–137.

19. Roy SA, Alloway KD (2001) Coincidence detection or temporal integration? What the neurons in somatosensory cortex are doing. J Neurosci 21: 2462–2473.
