# Supplementary figures and images for: Timescales of Multineuronal Activity Patterns Reflect Temporal Structure of Visual Stimuli
Source: PLoS One. 2011 Feb 8;6(2):e16758. doi: 10.1371/journal.pone.0016758 (PMC3035626; doi:10.1371/journal.pone.0016758)

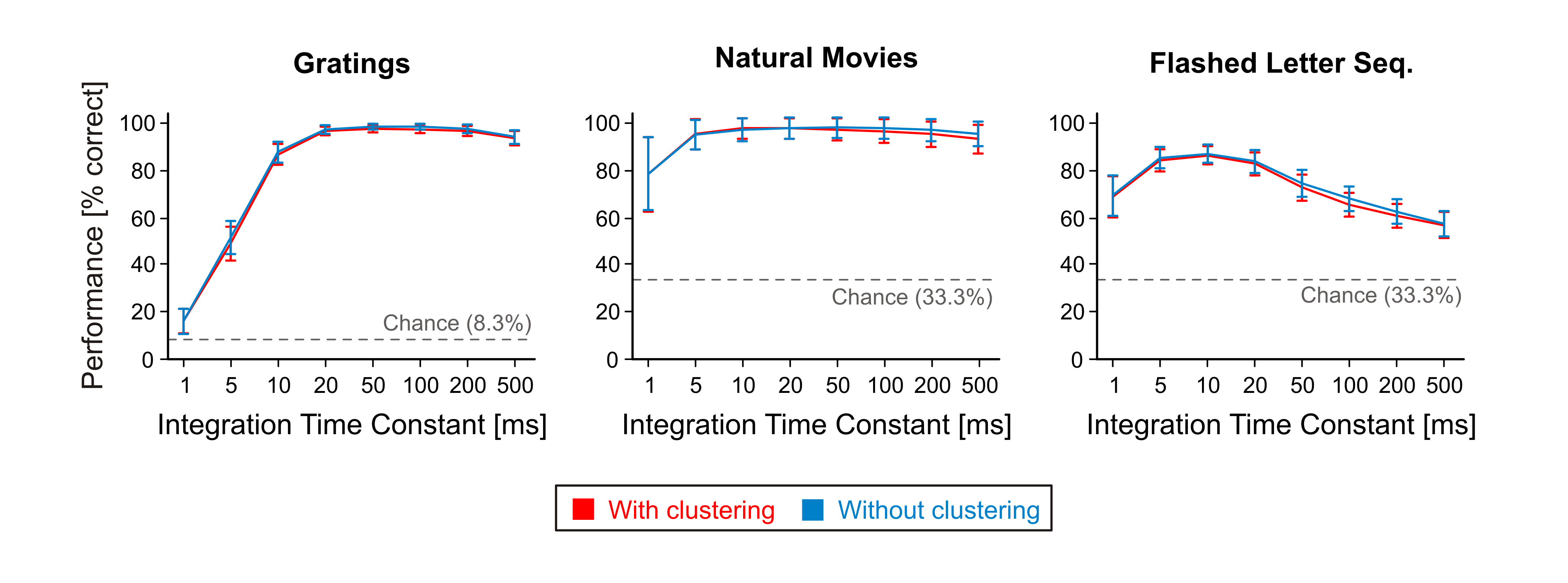

Supplement: Figure S1 — Classification performance of the trajectory classifier applied on data clustered with 3D Kohonen maps (red) and on unclustered data (blue). Error bars represent s.d. (TIF) [file pone.0016758.s002.tif]

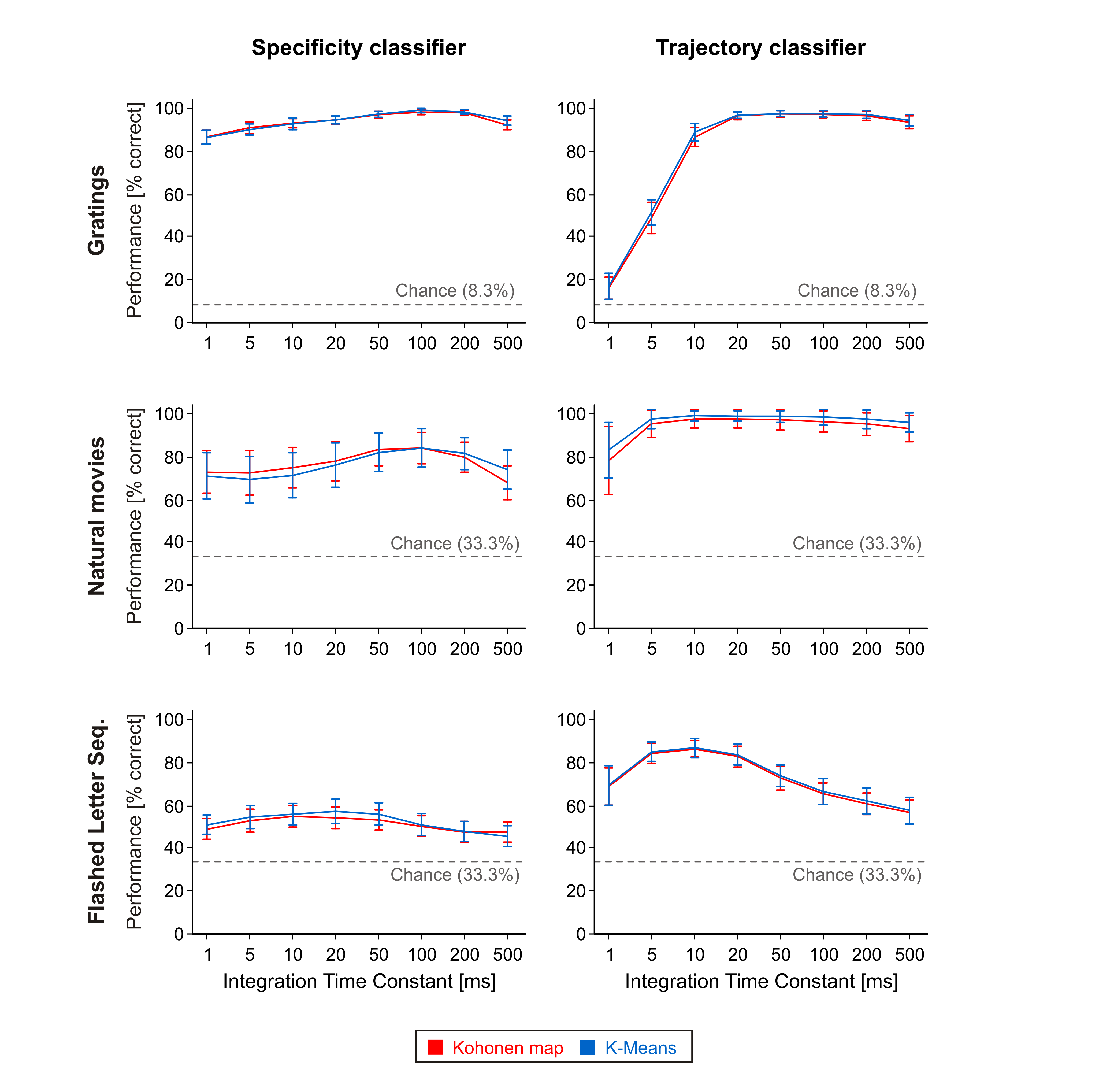

Supplement: Figure S2 — Classification performance of the specificity and trajectory classifiers applied on data clustered with 3D Kohonen maps (red) and with K-Means (blue). Error bars represent s.d. (TIF) [file pone.0016758.s003.tif]

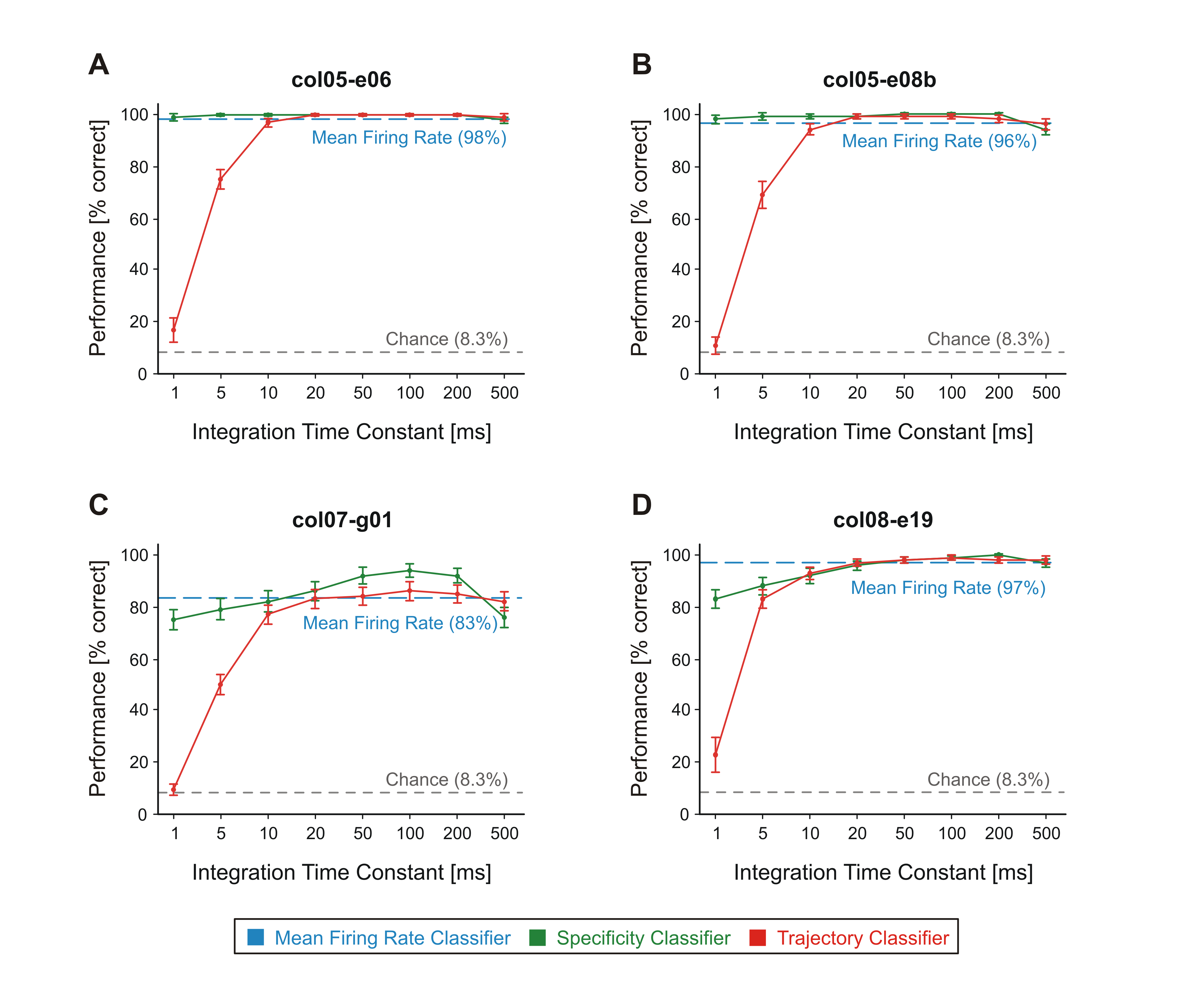

Supplement: Figure S3 — Reproduction of classification results for datasets evoked by drifting sinusoidal gratings. (A) and (B), Classification results for two datasets recorded from the same cat as in Figure 3D. The example in (B) is the same dataset as in Figure 3D but resorted according to different criteria. (C) and (D), Reproduction of classification results in two additional cats. Error bars represent s.d. (TIF) [file pone.0016758.s004.tif]

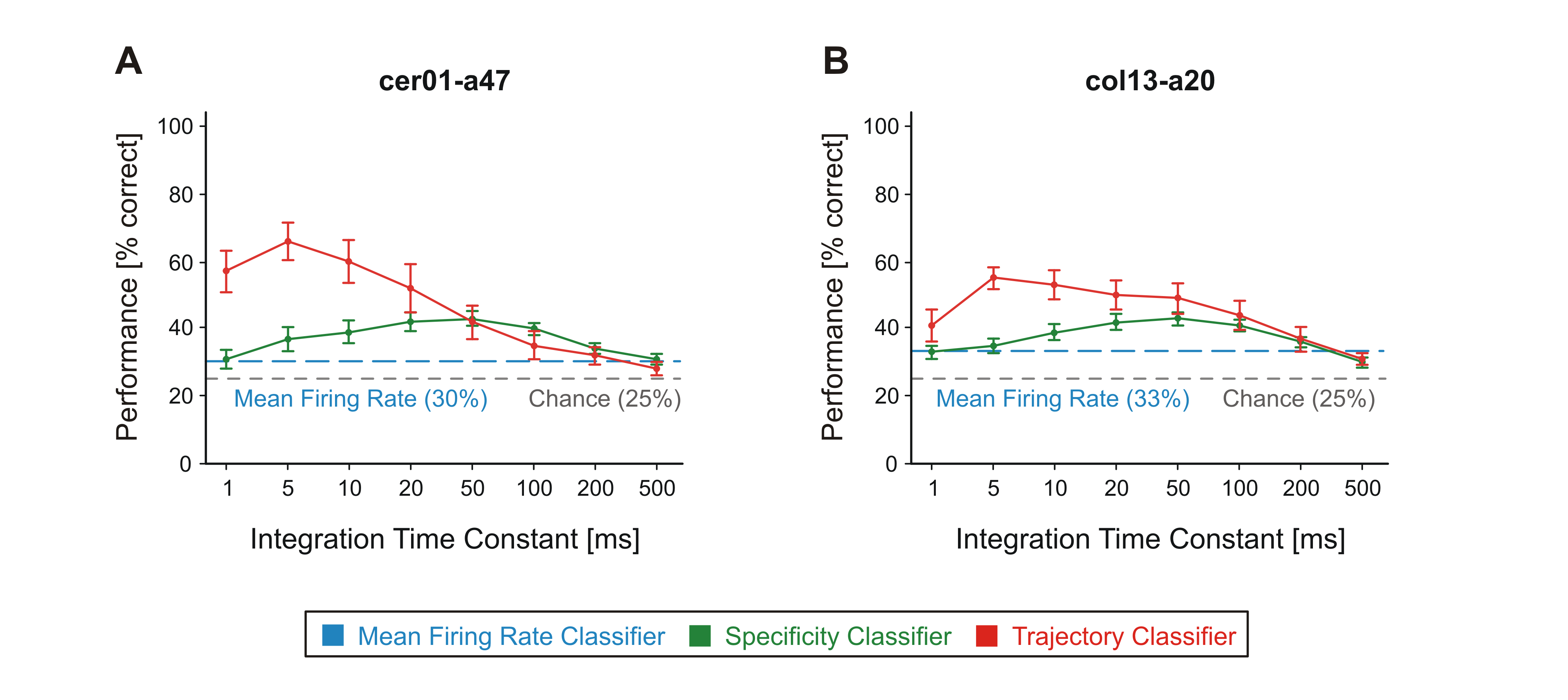

Supplement: Figure S4 — Reproduction of classification results from Figure 3F. (A) and (B), Results on datasets recorded in response to flashed letter sequences, from two additional cats. Error bars represent s.d. (TIF) [file pone.0016758.s005.tif]

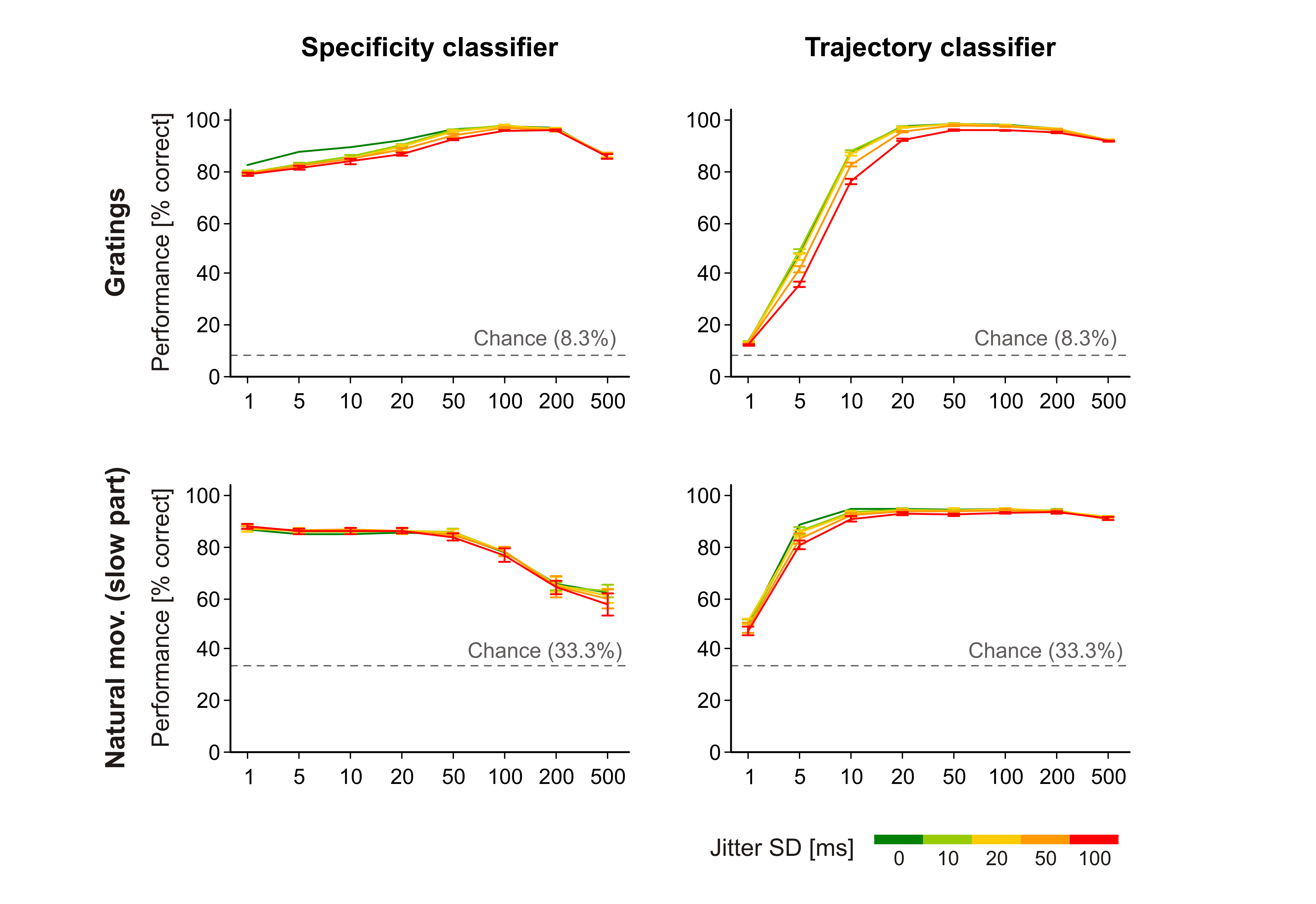

Supplement: Figure S5 — Effect of jitter on datasets with slow stimuli after bursts have been eliminated by keeping only the first spike in each burst. Error bars represent s.d. over independent jitters. (TIF) [file pone.0016758.s006.tif]
